# 3H-1,2-dithiole-3-thione protects retinal pigment epithelium cells against Ultra-violet radiation via activation of Akt-mTORC1-dependent Nrf2-HO-1 signaling

**DOI:** 10.1038/srep25525

**Published:** 2016-05-06

**Authors:** Ke-ran Li, Su-qing Yang, Yi-qing Gong, Hong Yang, Xiu-miao Li, Yu-xia Zhao, Jin Yao, Qin Jiang, Cong Cao

**Affiliations:** 1 Institute of Neuroscience, Soochow University, Suzhou, Jiangsu 215021, China; 2 The Affiliated Eye Hospital of Nanjing Medical University, Nanjing 210029, China

## Abstract

Excessive UV radiation and reactive oxygen species (ROS) cause retinal pigment epithelium (RPE) cell injuries. Nrf2 regulates transcriptional activation of many anti-oxidant genes. Here, we tested the potential role of 3H-1,2-dithiole-3-thione (D3T) against UV or ROS damages in cultured RPE cells (both primary cells and ARPE-19 line). We showed that D3T significantly inhibited UV-/H_2_O_2_-induced RPE cell death and apoptosis. UV-stimulated ROS production was dramatically inhibited by D3T pretreatment. D3T induced Nrf2 phosphorylation in cultured RPE cells, causing Nrf2 disassociation with KEAP1 and its subsequent nuclear accumulation. This led to expression of antioxidant response elements (ARE)-dependent gene heme oxygenase-1 (HO-1). Nrf2-HO-1 activation was required for D3T-mediated cytoprotective effect. Nrf2 shRNA knockdown or S40T dominant negative mutation as well as the HO-1 inhibitor Zinc protoporphyrin (ZnPP) largely inhibited D3T’s RPE cytoprotective effects against UV radiation. Yet, exogenous overexpression Nrf2 enhanced D3T’s activity in RPE cells. Further studies showed that D3T activated Akt/mTORC1 in cultured RPE cells. Akt-mTORC1 inhibitors, or Akt1 knockdown by shRNA, not only inhibited D3T-induced Nrf2-HO-1 activation, but also abolished the RPE cytoprotective effects. *In vivo*, D3T intravitreal injection protected from light-induced retinal dysfunctions in mice. Thus, D3T protects RPE cells from UV-induced damages via activation of Akt-mTORC1-Nrf2-HO-1 signaling axis.

Age-related macular degeneration (AMD) is a progressive degenerative retinal disease that would cause blindness if not handled properly^1^. The precise etiology of AMD is still not fully understood, although sunlight Ultra-violet (UV) radiation and subsequent reactive oxygen species (ROS) damages are recognized as the main contributors^1^,^2^,^3^. The link between oxidative stress and AMD is further supported by the results of clinical trial studies, which demonstrated a dramatic reduction AMD progression in subjects taking anti-oxidants and/or zinc-containing supplements^1^,^2^,^3^.

Vision loss among AMD patients starts from abnormalities in the retinal pigment epithelium (RPE), along with photoreceptor damages, Bruch’s membrane thickening, and choriocapillary hypo-perfusion^1^,^2^,^3^. These are considered as the main characteristics of AMD’s pathogenesis^1^. Following UV radiation, reactive oxygen species (ROS) including superoxide, hydroxyl radical, and singlet oxygen, as well as non-radical species such as hydrogen peroxide (H_2_O_2_) will be produced, which damage the cellular components of RPE cells, leading to RPE cell apoptotic death^4^,^5^. Our group has been studying the underlying mechanisms of UV/ROS-induced RPE cell injuries, and has explored the potential interfering strategies using *in vitro* systems^6^,^7^,^8^,^9^,^10^,^11^,^12^.

Antioxidant-responsive element (ARE) is a cis-acting regulatory element in the promoter region, which is critical for regulation of many genes encoding anti-oxidant proteins (i.e. heme oxygenase-1 (HO-1)) and phase II detoxification enzymes (i.e. NADPH)^13^,^14^. The NF-E2-related factor 2 (Nrf2) regulates transcriptional activation of above genes through binding to ARE^15^. Thus, Nrf2-ARE-mediated cytoprotective effect is thought to be dependent mainly on neutralization of oxidative stresses^13^. Thus, Nrf2-ARE is an important therapeutic target for oxidative stress prevention^13^,^14^. For example, our previous study has shown that Salvianolic acid A, the aqueous extract of the root of Salvia miltiorrhiza, protects RPE cells from H_2_O_2_ through activating Nrf2-HO-1 signaling^6^.

Dithiolethiones, the cyclic sulfur-containing compounds, are derived from cruciferous vegetables^16^,^17^. Existing evidences have demonstrated that dithiolethiones are able to efficiently induce production of antioxidants and phase II enzymes^16^,^17^, which are mediated mainly through activating Nrf2-ARE signaling^14^,^16^,^17^,^18^,^19^. Among all the dithiolethiones, 3H-1,2-dithiole-3-thione (D3T) is known as the most potent dithiolethione that activates Nrf2-ARE axis^16^,^19^. However, the detailed signaling mechanisms are still not fully understood. In the current study, we tested the potential role of D3T in UV-irradiated RPE cells, and studied the associated molecular mechanisms. The *in vivo* activity of D3T in mice was also analyzed.

## Results

### D3T inhibits UV-induced RPE cell death

**Figure 1:**
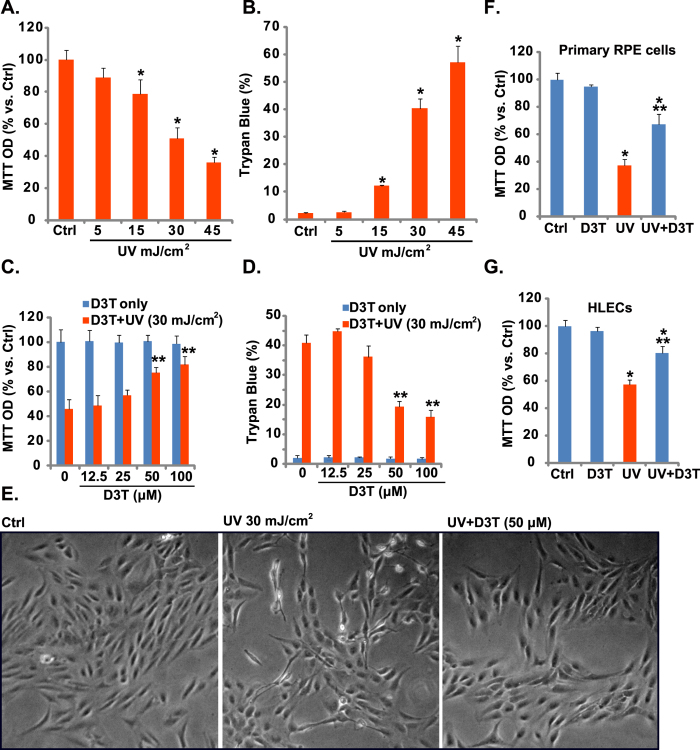
D3T inhibits UV-induced RPE cell damages. APRE-19 cells were either left untreated (“Ctrl”, for all figures), or irradiated with indicated intensity of UV, cells were further cultured for 24 h, cell viability was tested by MTT assay (**A**), and cell death was detected by trypan blue staining (**B**). APRE-19 cells were pre-treated with indicated concentrations of D3T (30 min pretreatment), followed by UV radiation (30 mJ/cm^2^), cells were further cultured for 24 h, cell viability and cell death were tested (**C**,**D**), representative morphology images were taken (**E**). Primary murine RPE cells and human HLECs cells were irradiated with UV (30 mJ/cm^2^), with or without D3T (50 μM, 30 min pretreatment), cells were further cultured for 24 h, and cell viability was tested (**F**,**G**). Experiments were repeated three times to insure consistency of results. *p < 0.05 vs. “Ctrl” group (**A**,**B**,**F**,**G**). **p < 0.05 vs. UV only group (**C**,**D**,**F**,**G**). Magnification: 1: 200 (**E**).

MTT results in Fig. 1A demonstrated that UV radiation dose-dependently inhibited human RPE cell (APRE-19 line^6^,^7^) survival. Further, the number of trypan blue positive RPE cells increased dramatically following with UV (15–45 mJ/cm^2^) radiation, indicating cell death (Fig. 1B). Significantly, D3T (50/100 μM) pretreatment (30 min) attenuated UV-induced RPE cell viability reduction (Fig. 1C) and cell death (Fig. 1D). Note that D3T itself, even at a very high dose (100 μM), had no detectable effect on RPE cell survival nor cell death (Fig. 1C,D). Phase contrast microscope images in Fig. 1E confirmed the cytoprotective effect of D3T against UV. In primary cultured murine RPE cells and human HLECs, D3T pretreatment similarly suppressed UV-induced viability reduction (Fig. 1F,G). Together, these results demonstrate that D3T inhibits UV-induced RPE cell death.

### D3T inhibits UV-induced RPE cell apoptosis

**Figure 2:**
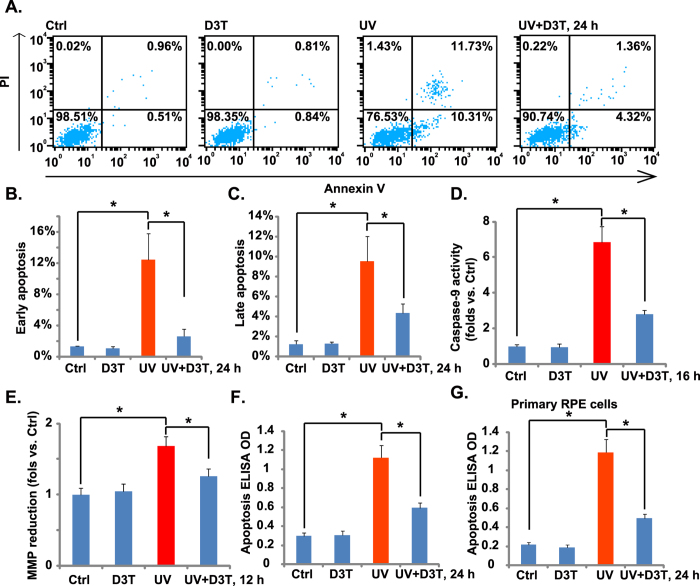
D3T inhibits UV-induced RPE cell apoptosis. APRE-19 cells (**A**–**F**) or primary murine RPE cells (**G**) were irradiated with UV (30 mJ/cm^2^) with or without D3T (50 μM, 30 min pretreatment), cells were further cultured for indicated time. Cell apoptosis was tested by PI-Annexin V FACS assay ((**A**–**C)**, for ARPE-19 cells) or histone-DNA apoptosis ELISA assay (**F**,**G**). Caspase-9 activity ((**D**) for ARPE-19 cells) and MMP reduction ((**E**) for ARPE-19 cells) were also shown. Experiments were repeated three times to insure consistency of results. *p < 0.05 (**B**–**G**).

Above results demonstrated that D3T inhibited UV-induced RPE cell death, next we tested the possible involvement of apoptosis in the process. RPE cell apoptosis was examined using the methods described^6^. FACS results in Fig. 2A demonstrated that UV (30 mJ/cm^2^) induced RPE cell apoptosis, with more than 10% of cells showing early apoptotic sign (PI^−/−^ and Annexin V^+/+^), and another 10% of cells with late apoptotic sign (PI^+/+^, Annexin V^+/+^) (Fig. 2B,C). Further, we showed that the caspase-9 activity was increased following UV irradiation in ARPE-19 cells (Fig. 2D). UV also induced mitochondrial membrane potential (MMP) reduction (Fig. 2E), which was tested by JC-10 dye assay^20^. These results indicated mitochondrial apoptosis pathway activation^21^,^22^ in UV-irradiated RPE cells. Notably, pretreatment with the caspase-9 inhibitor z-LEHD-fmk^23^ or the mPTP blocker sanglifehrin A (SfA)^24^ dramatically attenuated UV-induced apoptosis activation (Supplementary Fig. 1A,B). More importantly, UV irradiation-induced caspase-9 activation (Fig. 2D), MMP reduction (Fig. 2E) and subsequent cell apoptosis (Fig. 2A–C,F) were all attenuated with pre-treatment with D3T in ARPE-19 cells. These results suggested that D3T possibly inhibited UV irradiation-induced mitochondrial apoptosis pathway activation in RPE cells. On the other hand, we failed to observe significant caspase-8 activation in D3T-treated ARPE-19 cells (Data not shown). Activation of caspase-8 is a characteristic marker of extrinsic apoptosis pathway activation^21^,^22^. z-IETD-fmk, the capase-8 inhibitor^25^, showed almost no inhibitory effect on UV-induced ARPE-19 cell apoptosis (Supplementary Fig. 1A,B). In primary cultured murine RPE cells (Fig. 2G) and human HLECs (Data not shown), D3T pretreatment again significantly inhibited UV-induced apoptosis activation.

### D3T inhibits UV-induced ROS production, and protects RPE cells from H_2_O_2_

**Figure 3:**
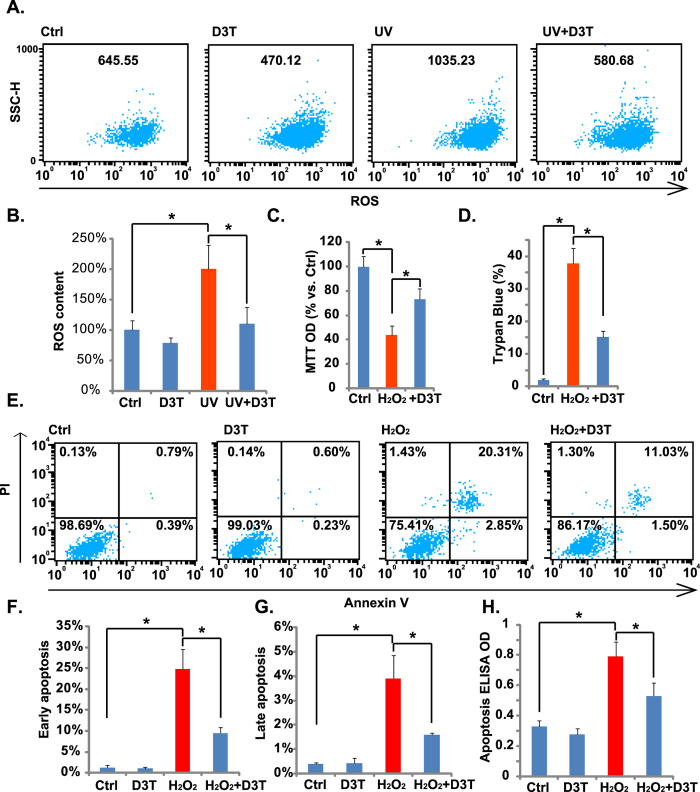
D3T inhibits UV-induced ROS production, and protects RPE cells from H_2_O_2_. APRE-19 cells were irradiated with UV (30 mJ/cm^2^) in the presence or absence of D3T (50 μM, 30 min pretreatment), ROS production was tested by FACS assay after 4 h (**A**,**B**). APRE-19 cells were treated with H_2_O_2_ (200 μM) in the presence or absence of D3T (50 μM, 30 min pretreatment), cells were further cultured for 24 h, and cell viability, cell death and apoptosis were analyzed by MTT assay (**C**), trypan blue staining (**D**) and PI-Annexin V FACS (**E**–**G**)/histone-DNA apoptosis ELISA assay (**H**), respectively. SSC-H: Side scatter (**A**, for ROS detection). Experiments were repeated three times to insure consistency of results. *p < 0.05 (**B**–**D**,**F**–**H**).

ROS production mediates UV-induced cell damages^26^. Above results showed that D3T protected RPE cells from UV. We then tested the effect of D3T on UV-induced ROS production. As shown in Fig. 3A,B, D3T (50 μM) pretreatment dramatically inhibited UV-induced ROS production in RPE cells. Based on these results, we proposed that D3T could protect RPE cells from direct reactive oxidative damages (i.e. H_2_O_2_). As a matter of fact, D3T suppressed H_2_O_2_-induced RPE cell viability reduction (Fig. 3C) and cell death (Fig. 3D). Meanwhile, H_2_O_2_-induced RPE cell apoptosis, tested by the Annexin V FACS assay (Fig. 3E–G) and histone-DNA apoptosis ELISA assay (Fig. 3H), was also inhibited by D3T pretreatment. These results demonstrate that D3T inhibits UV-induced ROS production, and protects RPE cells from H_2_O_2_.

### D3T activates Nrf2-HO-1 signaling in RPE cells

**Figure 4:**
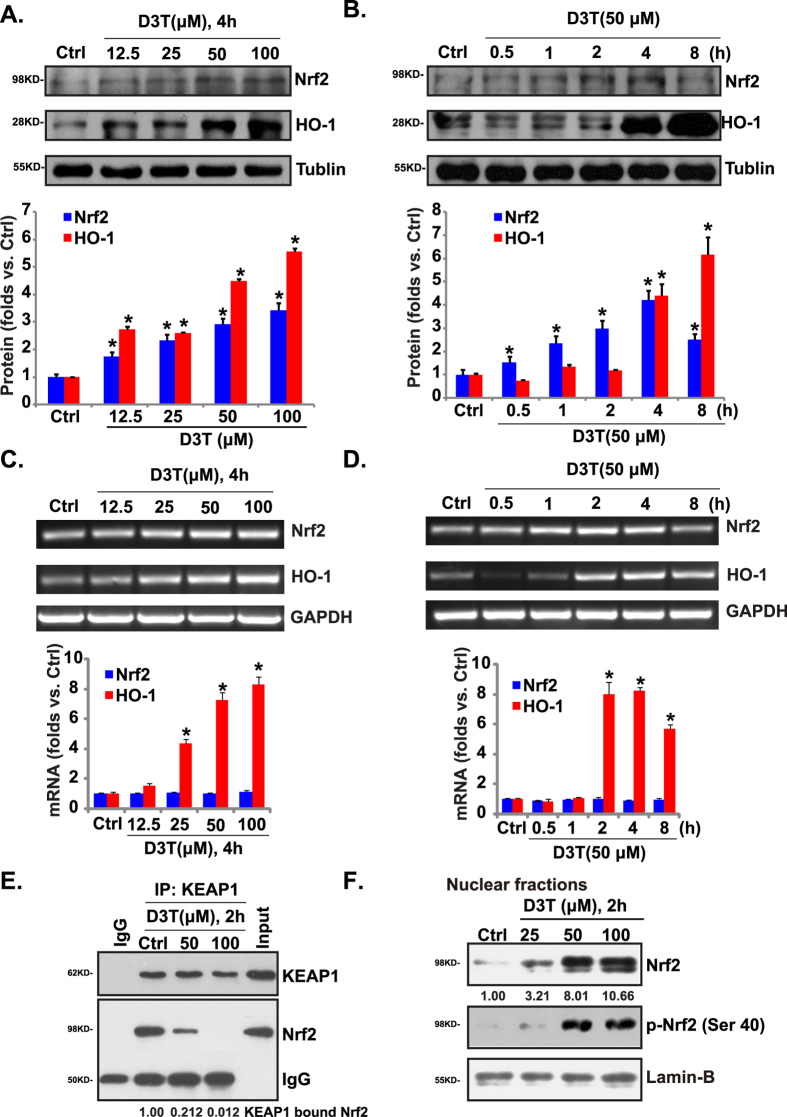
D3T activates Nrf2-HO-1 signaling in RPE cells. The protein and mRNA expressions of Nrf2 and HO-1 in APRE-19 cells with indicated D3T treatment were tested by Western blots (**A**,**B**) and RT-PCR (**C**,**D**). APRE-19 cells were treated with D3T (50/100 μM) for 2 h, the association between KEAP1 and Nrf2 was examined by Co-IP (**E**). Nrf2 (p- and regular) and Lamin-B expressions in the nuclei of APRE-19 cells with indicated D3T treatment were tested by Western blots (**F**). Experiments were repeated three times, and similar results were obtained. Each Western or PCR bands were quantified. *p < 0.05 vs. untreated control (“Ctrl”) group (**A**,**B**).

Results in Fig. 3 showed that D3T inhibited UV-induced ROS production, and protected RPE cells from oxidative stress. The Nrf2 and its regulated gene HO-1 are important components of the cellular anti-oxidant response^27^. HO-1 is an ARE-regulated gene, it is the rate-limiting enzyme for the conversion of heme into carbon monoxide (CO), free Fe^2+^, and biliverdin. Since D3T is a Nrf2 activator^28^,^29^,^30^, we tested Nrf2-HO-1 signaling in D3T-treated RPE cells. As shown in Fig. 4A,B, Nrf2 and HO-1 protein expressions were both increased following indicated D3T treatment. The effect of D3T on Nrf2-HO-1 expressions was dose- (Fig. 4A) and time-dependent (Fig. 4B). Significantly, the HO-1 mRNA expression was also increased following D3T treatment, while Nrf2 mRNA was almost unchanged (see quantification, Fig. 4C,D). mRNA levels of other phase II detoxification enzymes including NAD(P)H:quinone oxidase-1 (NQO-1), γ-glutamyl-cysteine ligase catalytic subunit (GCLC), and γ-glutamyl-cysteine ligase modifying subunit (GCLM)^31^ were also increased in D3T-treated ARPE-19 cells (Supplementary Fig. 2).

In the resting condition, KEAP1 represses Nrf2 activity by directly binding to Nrf2 N-terminal Neh2 domain, leading to Nrf2 cytosol sequestration and proteasomal degradation^32^. Activated Nrf2 (i.e. by phosphorylation) disassociates with KEAP1, leading to Nrf2 stabilization and accumulation in nuclei, where it induces transcription of HO-1 and other ARE genes^32^. Using the same Co-IP method as described^6^, we found that D3T disrupted Nrf2-KEAP1 association in cytosol (Fig. 4E). More importantly, when analyzing nuclear proteins^6^, we found that D3T induced Nrf2 phosphorylation and nuclear accumulation (Fig. 4F). Note that Nrf2 phosphorylation at Serine 40 is critical for its activation and stabilization^31^,^32^,^33^. These might explain upregulation of Nrf2 protein, but not Nrf2 mRNA, by D3T treatment (Fig. 4A–D). Together, these results show that D3T activates Nrf2-HO-1 signaling in ARPE-19 cells.

### Nrf2-HO-1 mediates D3T-induced RPE cytoprotective effect against UV

**Figure 5:**
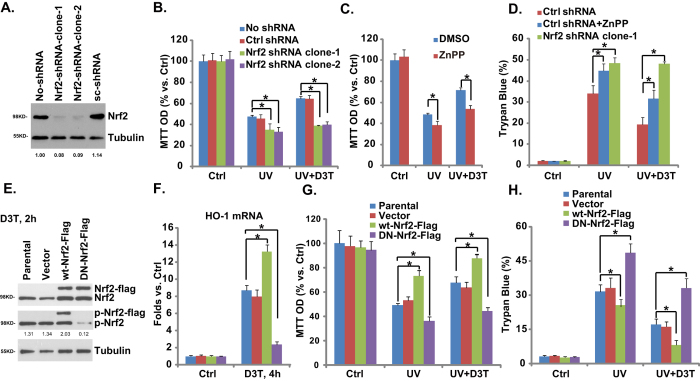
Nrf2-HO-1 mediates D3T-induced RPE cytoprotective effect against UV. Stable APRE-19 cells expressing scramble-shRNA (“Ctrl shRNA”) or Nrf2 shRNA (clone-1/-2) were radiated with UV (30 mJ/cm^2^), or together with D3T (50 μM, 30 min pretreatment), cells were further cultured for 24 h, Nrf2 and tubulin expressions were tested by Western blots, Nrf2 expression was quantified (**A**), cell viability and cell death were tested by MTT assay (**B**) and trypan blue staining (**D**), respectively. The effects of ZnPP (10 μM, 1 h pretreatment) on D3T (50 μM, 30 min pretreatment)-mediated protective effect against UV (30 mJ/cm^2^, cultured for 24 h) in APRE-19 cells were tested by MTT assay (**C**) and trypan blue staining (**D**). Stable ARPE-19 cells expressing dominant negative Nrf2 (S40T, DN-Nrf2), wild-type (wt-) Nrf2 or vector (pSV2 puro Flag), as well as the parental ARPE-19 cells were treated with D3T (50 μM) for indicated time, Nrf2 and tubulin expressions were tested by Western blots (**E**), Nrf2 phosphorylation (vs. Tubulin) was quantified (**E**); HO-1 mRNA expression was tested (**F**). Above ARPE-19 cells were radiated with UV (30 mJ/cm^2^), or together with D3T (50 μM, 30 min pretreatment), cells were further cultured for 24 h, cell survival and cell death were tested (**G**,**H**). Experiments were repeated three times, and similar results were obtained. *p < 0.05.

Above results have shown that D3T activates Nrf2-HO-1 signaling and protects against UV-induced RPE cell injuries. We then tested the link between the two. Using the same method described^6^, we applied Nrf2 shRNA-lentiviral particles to knockdown Nrf2, and resulting stable cells were selected by puromycin. Western blot results in Fig. 5A demonstrated that Nrf2 expression was dramatically downregulated in stable RPE cells expressing Nrf2-shRNA (both clone-1 and clone-2). Significantly, compared to the control cells, RPE cells with Nrf2-shRNA were more vulnerable to UV-induced damages (Fig. 5B,D). More importantly, D3T-mediated anti-UV effect was almost abolished with Nrf2 silencing (Fig. 5B,D). In consistent with these data, we found that zinc protoporphyrin (ZnPP), the HO-1 inhibitor, also inhibited D3T-induced RPE cytoprotective effects (Fig. 5C,D), note that ZnPP itself also intensified UV-induced RPE damages (Fig. 5C,D). These results suggest that Nrf2-HO-1 inhibition not only exacerbates UV-induced RPE cell damages, but also abolishes D3T-induced RPE cytoprotective activity against UV radiation.

To further confirm the role of Nrf2 in D3T-mediated actions, we exogenously introduced a dominant negative Nrf2 (S40T, DN-Nrf2) into RPE cells, and stable cells were selected (See methods). As expected, D3T-induced Nrf2 Ser-40 phosphorylation was abolished in DN-Nrf2 expressing cells (Fig. 5E). HO-1 expression by D3T was also dramatically inhibited (Fig. 5F). As a result, D3T-mediated RPE cytoprotection against UV was also inhibited in DN-Nrf2 expressing RPE cells (Fig. 5G,H). On the other hand, overexpression of wild-type (wt-) Nrf2 facilitated D3T-induced Nrf2 Ser-40 phosphorylation (Fig. 5E) and HO-1 expression (Fig. 5F). RPE cytoprotection by D3T was also enhanced in Nrf2-overexpressed RPE cells (Fig. 5G,H). Note that wt-Nrf2 overexpression by itself also inhibited UV damages in RPE cells (Fig. 5G,H). These results again indicate that Nrf2 Ser-40 phosphorylation is important for D3T-induced Nrf2 activation and RPE cytoprotection.

### Activation of Akt-mTORC1 is required for D3T-induced Nrf2-HO-1 activation and RPE cytoprotection

**Figure 6:**
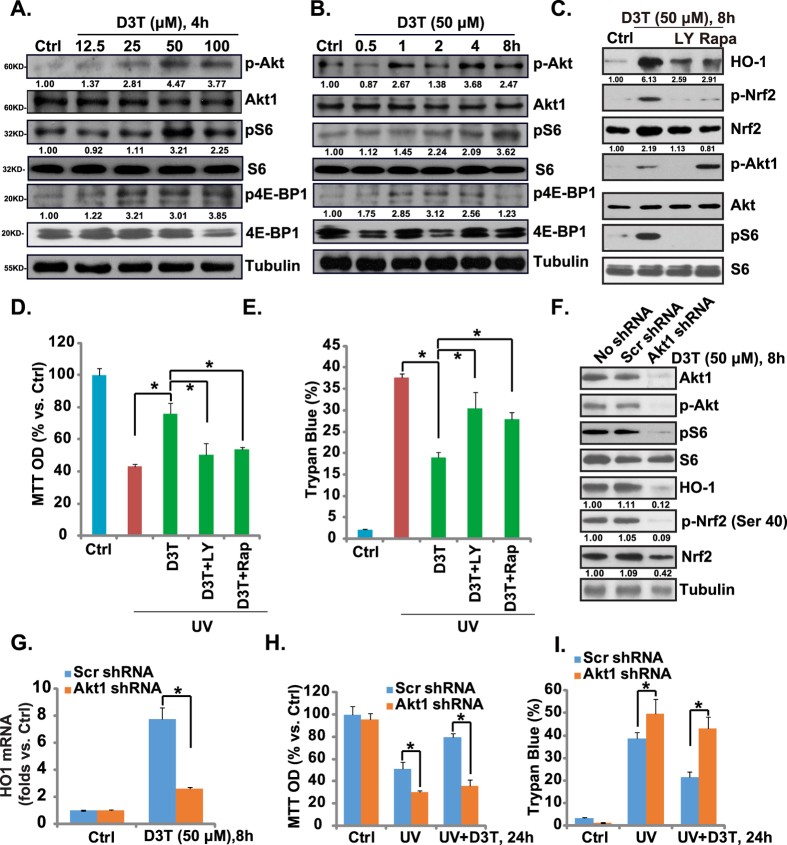
Activation of Akt-mTORC1 is required for D3T-induced Nrf2-HO-1 activation and RPE cytoprotection. APRE-19 cells were treated with indicated concentrations of D3T, and cultured for indicated time, p-Akt (Ser 473), Akt, p-S6 (Ser 235/236), S6, p-4E-BP1 (Ser 65), 4E-BP1 and tubulin were tested by Western blots, kinase phosphorylations were quantified (**A**,**B**). APRE-19 cells were pre-treated with LY294002 (500 nM) or rapamycin (100 nM) for 1 h, followed by UV (30 mJ/cm^2^), or with D3T (50 μM, 30 min pretreatment), cell were further cultured, Western blots were utilized to test indicated proteins (**C**), while cell viability and cell death were tested 24 h after UV treatment (**D**,**E**). Stable APRE-19 cells expressing scramble-shRNA or Akt1 shRNA were radiated with UV (30 mJ/cm^2^), and/or D3T (50 μM, 30 min pretreatment), cells were further cultured, expressions of listed proteins were tested by Western blots, and quantified (**F**), HO-1 mRNA expression was tested (**G**), cell survival and cell death were also tested by MTT assay (**H**) and Trypan blue staining assay (**I**). Experiments were repeated three times, and similar results were obtained. *p < 0.05.

Our previous study has shown that Salvianolic acid A activated Nrf2 through phosphorylation in a Akt-mTORC1-dependent manner, and Nrf2-HO-1 activation by Salvianolic acid A was blocked by Akt-mTORC1 inhibitors^6^. In the current study, we found that D3T activated Akt-mTORC1 signaling in cultured RPE cells, and the effect of D3T was both time- and dose-dependent (Fig. 6A,B). Note that Akt activation was shown by upregulation of p-Akt (Ser 473), while mTORC1 activation was reflected by phosphorylations of S6 (Ser S235/236) and 4E-BP1 (Ser 65) (Fig. 6A,B). Significantly, LY294002, the broad PI3K/Akt/mTOR inhibitor^34^, as well as the mTORC1 inhibitor rapamycin, strongly inhibited D3T-induced Nrf2 phosphorylation and accumulation as well as HO-1 induction (Fig. 6C), indicating that Akt-mTORC1 activation was involved in D3T-mediated activation of Nrf2-HO-1 cascade. Functionally, LY294002 and rapamycin attenuated D3T-induced RPE cytoprotective effect against UV (Fig. 6D,E).

Next, Akt1 shRNA containing lentiviral particles were applied to knockdown Akt1, and stable RPE cells were selected. As shown in Fig. 6F, Akt1 expression was dramatically downregulated in stable cells. As a result, D3T-induced Akt and S6 phosphorylations, Nrf2 phosphorylation (Ser 40) and accumulation, as well as HO-1 protein or mRNA expressions were largely inhibited (Fig. 6F,G). Akt1-knockdown RPE cells were more sensitive to UV damages (Fig. 6H,I). More importantly, D3T-mediated RPE cytoprotective effect was almost completely abolished with Akt1 knockdown. These results confirmed a critical role of Akt-mTORC1 signaling in D3T-induced Nrf2-HO-1 activation and RPE cytoprotection.

Note that UV itself induced mild Akt-HO-1 activation in ARPE-19 cells (Supplementary Fig. 3A,B). That might explain why Nrf2-silenced/-mutated ARPE-19 cells were slightly more vulnerable to UV irradiation than the control cells (Fig. 5). UV-induced Akt-HO-1 activation was much weaker than that by D3T (Supplementary Fig. 3A,B). Significantly, D3T and UV combination didn’t show an additive effect on Akt-HO-1 activation (Supplementary Fig. 3A,B). In another word, UV-induced Akt-HO-1 activation might just be negligible when D3T was present.

### D3T activates Akt/mTORC and Nrf2-HO-1 signalings in primary murine RPE cells

**Figure 7:**
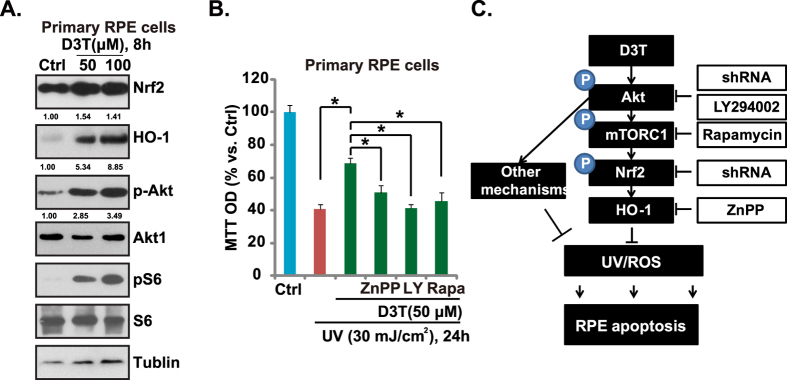
D3T activates Akt-mTORC1 and Nrf2-HO-1 signalings in primary murine RPE cells. Primary cultured murine RPE cells were treated with indicated concentrations of D3T for 8 h, expression of indicated proteins was tested by Western blots (**A**). Primary murine RPE cells were pre-treated with D3T(50 μM, 30 min), or together with LY294002 (500 nM)/rapamycin (200 nM)/ZnPP (10 μM), followed by UV (30 mJ/cm^2^) radiation, cells were further cultured for 24 h before cell viability was tested (**B**). (**C**). The proposed signaling pathway of this study: D3T inhibits UV-induced RPE cell apoptosis partly through activating of Nrf2-HO-1 signaling axis and inhibiting ROS production. Akt-mTORC1 activation is important for D3T-induced Nrf2-HO-1 activation and RPE cytoprotective effect against UV. Experiments were repeated three times, and similar results were obtained. *p < 0.05.

Above results demonstrated that D3T induced Nrf2-HO-1 activation, which was mediated, at least in part, by activating Akt-mTORC1 signaling in human PRE cells. Next, we examined whether similar molecular events were also happening in the primary cultured murine RPE cells. Western blot results in Fig. 7A confirmed that D3T activated Akt/mTORC and Nrf2-HO-1 signalings in primary cultured murine RPE cells. Further, as shown in Fig. 7B, HO-1 inhibitor ZnPP, Akt-mTORC1 inhibitor LY294002 and mTORC1 inhibitor rapamycin inhibited the pro-survival effect of D3T against UV radiation in primary cultured RPE cells. Thus, D3T activates Akt-mTORC1 and Nrf2-HO-1 signalings, which mediate UV protective functions in primary murine RPE cells (Fig. 7C).

### D3T protects from light-induced retinal dysfunction *in vivo*

**Figure 8:**
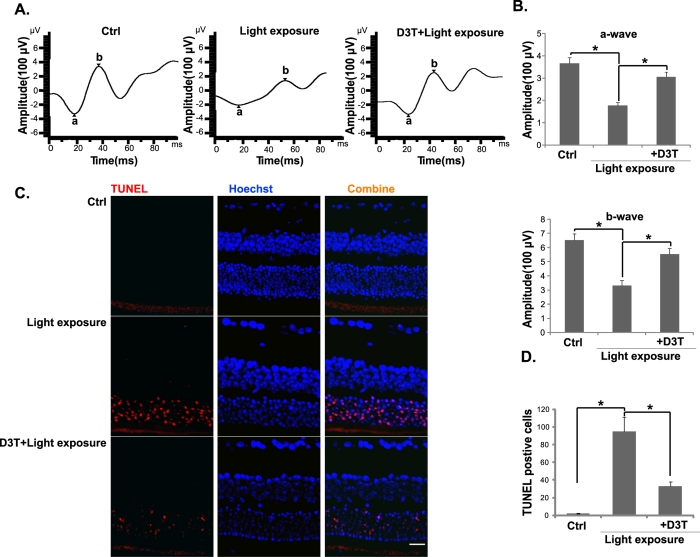
Effect of D3T on light-induced retinal dysfunctions in mice. ERG was measured 24 h after light exposure, represent amplitudes of a- and b-waves were shown (**A)**. Data were quantified (**B**). Retinal samples were also subjected to TUNEL staining, and representative TUNEL fluorescence images were shown (**C**). TUNEL data were also quantified (**D**). D3T treatment was at 10 mg/kg body weight 30 min before light exposure. For each analysis, n = 6. Experiments were repeated three times, and similar results were obtained. *p < 0.05. Scale bar, 50 μm (**C**).

Finally, we tested the effect of D3T on light-induced retinal dysfunction *in vivo*. ERG analysis was first applied. It has been shown that ERG dysfunction reached a peak 24 h after light exposure^35^. Therefore, this time point was chosen to evaluate D3T’s efficacy. Compared to the control mice, the amplitudes of ERG’s a- and b-waves were markedly decreased in light-exposed mice (Fig. 8A,B). D3T intravitreal injection (at 10 mg/kg body weight, 30 min pre-treatment) significantly attenuated light-induced decrease in a- and b-wave amplitudes (Fig. 8A,B). Further, retinal TUNEL staining assay results showed that light exposure induced significant apoptosis in outer nuclear layer (ONL) of mice retina, which was also suppressed by pre-injection of D3T (Fig. 8C,D). D3T injection alone showed no significant effect on the amplitudes of a- and b-wave, nor on retinal apoptosis (Data not shown). We also failed to detect any apparent deleterious effects by D3T to mice eyes. These results indicate that D3T protects from light-induced retinal damages *in vivo*.

## Discussions

Nrf2 plays a key regulatory role in expression of ARE-controlled phase II detoxifying and many important antioxidant enzymes, counteracting cellular oxidative stresses^27^. Although D3T has long been considered as an effective activator of Nrf2, the underlying signaling mechanisms are not fully addressed. Studies have shown that D3T interaction with the sulfhydryl groups of KEAP1 causes KEAP1-Nrf2 dissociation, Nrf2 stabilization and nuclear accumulation^36^. In the current study, we proposed a novel mechanism of D3T-induced Nrf2 activation: D3T activates Akt-mTORC1 signaling to phosphorylate Nrf2 (Ser 40), which is then apart from KEAP1, causing it stabilization and translocation to nuclei, where it promotes transcription of ARE-regulated genes (i.e. HO-1) (Fig. 7C). Our hypothesis is supported by the fact that Akt-mTORC1 inhibitors (LY294002 and rapamycin), or Akt1 shRNA knockdown, largely inhibited D3T-induced Nrf2 phosphorylation and nuclear accumulation as well as HO-1 upregulation.

Several signaling kinases including Erk, p38 MAPKs and PKC have been proposed to modify Nrf2 activity and enhance its translocation into the nuclei^27^. Recent studies including ours have suggested that PI3K/Akt and its downstream mTORC1 signaling is important mediator for Nrf2 activation. Lee *et al.*, showed that the Nrf2 activator sulforaphane activates Nrf2 through phosphorylation in a PI3K/Akt dependent manner, while LY294002 dramatically inhibited sulforaphane-induced Nrf2-HO-1 activation^37^. Similarly, pyocyanin is shown to activate Nrf2 though PI3K activation, and PI3K in-activation markedly reduced pyocyanin-stimulated Nrf2 nuclear accumulation as well as the transcriptional activation of ARE-genes^38^. Our recent study showed that Salvianolic acid A-induced Nrf2-HO-1 signaling activation is also mediated through Akt-mTORC1 signaling^6^. Here, we propose that rapamycin-sensitive mTOR signaling (mTORC1) is important for D3T-mediated Nrf2 phosphorylation/activation, which is supported by fact that rapamycin blocked D3T-induced Nrf2 phosphorylation/accumulation and HO-1 expression.

In this study, we showed that D3T-mediated Nrf2-HO-1 activation and its RPE cytoprotective effect against UV radiation were suppressed by Akt-mTORC1 inhibitors or Akt1 shRNA knockdown, suggesting that Akt-mTORC1 was required for D3T-mediated above actions. These results are consistent with our previous findings showing that nerve growth factor (NGF)-mediated RPE cytoprotective effect was also dependent on Akt-mTORC1 signaling^11^. Recently, we showed that α-melanocyte stimulating hormone (α-MSH) activated Akt-mTORC1 signaling to protect RPE cells against H_2_O_2_ damages^8^. These results are not surprising, as Akt-mTORC1 is a key pro-survival signaling^11^,^39^.

Existing evidences have shown that Ser-40 phosphorylation is critical for Nrf2 activation and stabilization^31^,^32^,^33^. In the current study, we show that D3T induces Nrf2 Ser-40 phosphorylation in an Akt-mTORC1 dependent manner, which appears required for subsequent Nrf2 activation. Nrf2 S40T mutation, which abolished D3T-induced Ser-40 phosphorylation, remarkably inhibited D3T-mediated HO-1 expression and RPE cytoprotection. On the other hand, overexpression of wt-Nrf2 could facilitate Nrf2 Ser-40 phosphorylation by D3T, leading to increased Nrf2 activation (HO-1 expression) and superior RPE cytoprotection. The detailed mechanisms underlying Nrf2 Ser-40 phosphorylation by D3T warrants further investigations.

In summary, the results of this study show that D3T protects RPE cells against UV radiation through activation of Nrf2-HO-1 signaling. Akt-mTORC1 activation appears required for D3T-induced Nrf2-HO-1 activation and RPE cytoprotective effects. Importantly, our *in vitro* studies showed that D3T intravitreal injection protected from light-induced retinal dysfunctions in mice. These results imply that D3T and possible other Nrf2 activators might have therapeutic values for AMD.

## Methods

### Ethics

All methods listed in the study were carried out in accordance with the approved guidelines by authors’ institutions (Nanjing Medical University and Soochow University).

### Reagents, chemicals and antibodies

D3T, H_2_O_2_ z-LEHD-fmk, z-IETD-fmk, sanglifehrin A (SfA) and Zinc protoporphyrin (ZnPP, the HO-1 inhibitor) were obtained from Sigma (St. Louis, MO). Rapamycin and LY294002 were purchased from Calbiochem (Darmstadt, Germany). The antibody against tubulin was purchased from Sigma (St. Louis, MO). KEAP1, Nrf2 and HO-1 antibodies were purchased from Santa Cruz Biotech (Santa Cruz, CA). P-Nrf2 (Ser 40) antibody was purchased from Abcam (Shanghai, China). All other antibodies utilized in this study were described previously^6^.

### Cell culture

Human retinal pigment epithelial cells (ARPE-19 line) and human lens epithelial cells (HLECs) were cultured as previously described^40^,^41^.

### Primary murine RPE cells isolation and culture

As previously reported^8^,^10^, C57/B6 mice at age of 3-4 weeks were anesthesia by 75% alcohol, and the eyeballs in asepsis were taken out and diluted for several times with D-hank’s fluid. After soaking in the DMEM/F-12 (Hyclone) for 6-10 h, the eyeballs were taken out and the retinas were striped carefully. Parenzyme (0.125%) was then added to digest for 20 min at 37 °C before adding culture medium containing 10% FBS to terminate digestion. Then the supernatants were centrifuged twice at 1000 r/min in the culture medium (80% DMEM/F-12, 20% FBS) to produce cell suspension. The experiments were performed in accordance with the Institutional Animal Care and Use Committee (IACUC) and Association for the Assessment and Accreditation of Laboratory Animal Care (AAALAC). The protocols were approved by authors’ institutions (Nanjing Medical University and Soochow University).

### UV radiation

UV radiation (UVB and UVA2) to cultured cells was performed as previously reported^10^,^12^. Cells were irradiated at a desired intensity. Afterwards, cells were returned for incubation in basal medium with indicated treatments.

### Cell viability assay and morphology examination

As reported^6^, the cell viability was assessed using the MTT assay. The Optical Density (OD) value of treatment group was normalized to that of untreated control group^6^. The morphological change was observed under microscope (1*200), and photomicrographs were taken with Olympus digital camera (Shanghai, China).

### Trypan blue staining of “dead” RPE cells

After applied treatment, the “dead” RPE cells were stained with trypan blue, and the percentage (%) of dead cells was calculated by the number of the trypan blue stained cells divided by the total cell number, which was automatically recorded by an automated cell counter (Merck Millipore, Shanghai, China).

### ROS assay

As previously reported^6^,^7^, the ROS level was determined by carboxy-H2DCFDA (Calbiochem)-FACS assay. Briefly, after applied treatment, RPE cells were incubated with 1 μM of carboxy-H2-DCFDA at 37 °C for 30 min. Cells (1*10^6^) were then resuspended in PBS, and sent to flow cytometry analysis (BD bioscience).

### RT-PCR Analysis

As previously reported^6^, the total RNA was extracted using Trizol reagent (Invitrogen). Five hundred ng of DNA-free total RNA was used to perform the reverse transcription with the 2-step RT-PCR kit (Takara Bio Inc., Japan)^6^. The transcribed cDNA was utilized for PCR amplification with specific primers summarized in previous publication^6^. The primers for NAD(P)H:quinone oxidase-1 (NQO-1) were: Forward, 5′-CCATTCTGAAAGGCTGGTTTG-3′, and reverse: 5′-CTAGCTTTGATCTGGTTGTC-3′ 42. The primers for γ-glutamyl-cysteine ligase catalytic subunit (GCLC) were: Forward 5′-TTACCGAGGCTACGTGTCAGAC-3′ and reverse 5′-TGTCGATGGTCAGGTCGATGTC-3′; The primers for γ-glutamyl-cysteine ligase modifying subunit (GCLM) were: Forward 5′-AATCAGCCCTGATTTGGTCAGG-3′ and reverse 5′-CCAGCTGTGCAACTCCAAGGAC-3′ 43. PCR products were separated on 1.2% agarose gels and visualized with ethidium bromide (EB). Glyceraldehyde-3-phosphate dehydrogenase (GAPDH) was tested as an internal control.

### Western blots

Western blots were performed according to our previous protocol^6^,^10^,^11^. Each indicated band was quantified and normalized to the corresponding loading control through ImageJ software^10^. For detection of nuclear proteins, the nuclei of cultured RPE cells were isolated by the nuclei Isolation kit purchased from Sigma^6^.

### Co-immunoprecipitation (Co-IP) assay

As previously described^6^, KEAP-1-Nrf2 association was tested by Co-IP. Briefly, after treatment, RPE cells were lysed with ice-cold IP buffer A described^6^. To the cleared lysates, 2 μg of KEAP1 antibody was added per 1.2 mg of soluble proteins, and the immune complex was allowed to form by incubating with rotation for 24 h at 4 °C. The 50% slurry (25 μl) of protein A/G-Sepharose was then added and the incubation continued for 3 h. The resulting immuno-precipitates captured with protein G-Sepharose (Sigma) were washed four times with CHAPS-containing buffer and analyzed by Western blots.

### Apoptosis assay by Annexin-V FACS

After indicated treatment, the cell apoptosis was determined by the Annexin-V FACS assay according to the manufacturer’s protocol (Calbiochem). Both early apoptotic (Annexin V^+/+^/ PI^−/−^) and later apoptotic (Annexin V^+/+^/ PI^+/+^) RPE cells were gated^6^.

### Apoptosis assay by enzyme-linked immunosorbent assay (ELISA)

As previously reported^6^, the Cell Apoptosis histone-DNA ELISA Detection PLUS Kit (Roche, Palo Alto, CA) was utilized to quantify cell apoptosis.

### Detection of mitochondrial membrane potential (MMP)

As described in our previous study^20^, the cell MMP, an indicator of mitochondrial apoptosis pathway activation, was measured via JC-10 dye (Invitrogen, Shanghai, China). With the MMP decreasing, monomeric JC-10 will be formed in the cytosol, exhibiting green fluorescence. Briefly, after treatment, cells were stained with 5 μg/mL of JC-10 for 10 min at 37 °C. Afterwards, cells were washed twice with PBS, and resuspended in fresh culture medium and read immediately on a microplate reader with an excitation filter of 485 nm and emission filter of 527 nm. Fluorescence intensity was recorded as the indicator of MMP reduction (ΔΨm).

### Determination of caspase-8/-9 activity

After treatment, cell lysates were incubated with caspase-specific tetrapeptide substrates, which were labeled with *p*-nitroaniline (pNA). For caspase-8, IETD-*p*NA and caspase-9, LEHD-*p*NA were utilized (Biomol, Plymouth Meeting, PA). The lysates were incubated with each substrate for 30 min, and the absorbance was determined by a microplate reader (BioTek, Shanghai, China).

### ShRNA knockdown and stable cell selection

As previously described^6^, the lentiviral particles containing scramble control shRNA, Nrf2 shRNA (sc-37030-V) or Akt1 shRNA (sc-29195-V) were purchased from Santa Cruz Biotech (Santa Cruz); The lentiviral particles (10 μl/ml) were added to ARPE-19 cells for 36 h, and stable clones expressing corresponding shRNA were selected by puromycin (1.0 μg/ml). The puromycin-containing culture medium was renewed every 48 h, until resistant colonies can be identified (8–10 passages). The expressions of targeted protein and the loading control in stable cells were verified by Western blots.

### Nrf2 mutation or over-expression

The full-length Nrf2 cDNA covering Ser-40 (Genechem, Shanghai, China) was utilized as the template for generating the Nrf2 S40T cDNA. The S40T Nrf2 pSV2 puro Flag plasmid and the wild-type (wt−) Nrf2 pSV2 puro Flag plasmid were synthesized and verified by Genechem. The plasmid or the empty vector (pSV2 neo) was transfected into ARPE-19 cells with the Lipofectamine 2000 protocol (Invitrogen). After 48 h, ARPE-19 cells were re-plated on selection medium containing 2.5 μg/mL of puromycin for 12–14 days. Stable colonies were isolated, and characterized for expression of Nrf2 (Flag-tagged).

### Experimental animals and light damage

Two-month-old, weight-matched male BALB/c mice were selected. Light exposure was performed as previously described^35^. The mice were kept in total darkness for 24 h. One hour before light exposure, one drop of 0.5% tropicamide and 0.5% phenylephrine hydrochloride (Sigma, Shanghai, China) were applied to the cornea for pupillary dilation. The mice were then exposed to 5000 lux of white fluorescent light^35^. To test the potential activity of D3T *in vivo*, 30 min before light exposure, D3T (at 10 mg/kg body weight) were injected intravitreally to the left eyes. All studies were performed in accordance with the standards of ethical approval by all authors’ institutions. The protocols were also approved by the IACUC and international regulations.

### Electroretinography (ERG)

As described^35^, twenty-four h after light exposure, a single light-flash stimulus (3000 cd/m^2^ for 10 ms) was applied to the eye via a commercial system (EP1000 Electrophysiology; Tomey corporations, Japan). This ERG recording process was conducted under dim red light, and the mice were kept warm during the process. The b-wave amplitude was measured from the trough of the a-wave to the peak of the b-wave, and the amplitude of the a-wave was measured from the initial baseline^35^.

### Retinal TUNEL staining

Twenty-four h after light exposure, the enucleated eyes were fixed in 4% paraformaldehyde. After dehydration in a graded ethanol series, these samples were embedded in paraffin. Cryosections were cut in the sagittal plane through the optic nerve head. The sections were stained via terminal deoxynucleotidyl transferase (TdT)-mediated dUTP nick-end labeling (TUNEL) *in situ* cell death detection kit (Roche Diagnostics, Shanghai, China). The fluorescence image was taken via the Olympus IX-73 fluorescence microscope (Olympus, Tokyo, Japan).

### Statistical analysis

All data were normalized to the control values of each assay, and were presented as mean ± standard deviation (SD). Data were analyzed by one-way ANOVA followed by a Scheffe’s f-test by using SPSS 18.0 software (SPSS Inc., Chicago, IL). Significance was chosen as *p* < 0.05.

## Additional Information

**How to cite this article**: Li, K.-r. *et al.* 3H-1,2-dithiole-3-thione protects retinal pigment epithelium cells against Ultra-violet radiation via activation of Akt-mTORC1-dependent Nrf2-HO-1 signaling. *Sci. Rep.* **6**, 25525; doi: 10.1038/srep25525 (2016).

## Supplementary Material

Supplementary Information
