# Supplementary material for: 3H-1,2-dithiole-3-thione protects retinal pigment epithelium cells against Ultra-violet radiation via activation of Akt-mTORC1-dependent Nrf2-HO-1 signaling
Source: Sci Rep. 2016 May 6;6:25525. doi: 10.1038/srep25525 (PMC4858705; doi:10.1038/srep25525)
Supplement: Supplementary Information [file srep25525-s1.pdf]

The supplementary figures of the manuscript:

3H-1,2-dithiole-3-thione protects retinal pigment epithelium cells against Ultra-violet radiation via activation of Akt-mTORC1-dependent Nrf2-HO-1 signaling

Ke-ran Li, Su-qing Yang, Yi-qing Gong, Hong Yang, Xiu-miao Li, Yu-xia Zhao, Jin Yao, Qin Jiang and Cong Cao

Supplementary Fig. 1. of "*3H-1,2-dithiole-3-thione protects retinal pigment epithelium cells against Ultra-violet radiation via activation of Akt-mTORC1-dependent Nrf2-HO-1 signaling*" by Ke-ran Li, Su-qing Yang, Yi-qing Gong, Hong Yang, Xiu-miao Li, Yu-xia Zhao, Jin Yao, Qin Jiang and Cong Cao.

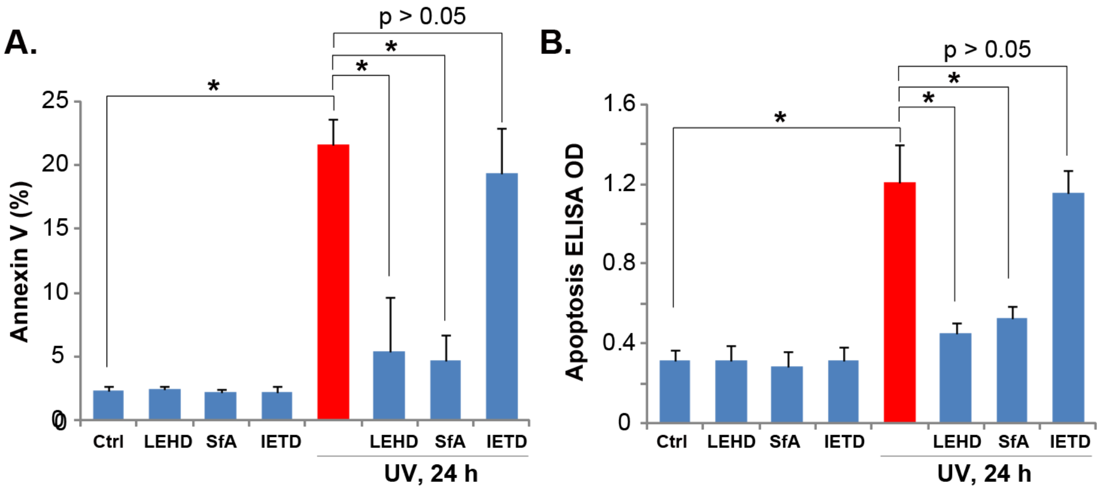

Supplementary Fig. 1. APRE-19 cells were pre-treated with the caspae-9 specific inhibitor z-LEHD-fmk ("LEHD", 50 μM), and the caspae-8 specific inhibitor z-IETD-fmk (50 μM) or sanglifehrin A (SfA, 1.0 μM) for 30 min, followed by UV (30 mJ/cm^2) irritation, cells were further cultured for 24 h. Cell apoptosis was tested by PI-Annexin V FACS assay (**A**) and histone-DNA apoptosis ELISA assay (**B**). Experiments were repeated three times to insure consistency of results. *p<0.05.

Supplementary Fig. 2. of "*3H-1,2-dithiole-3-thione protects retinal pigment epithelium cells against Ultra-violet radiation via activation of Akt-mTORC1-dependent Nrf2-HO-1 signaling*" by Ke-ran Li, Su-qing Yang, Yi-qing Gong, Hong Yang, Xiu-miao Li, Yu-xia Zhao, Jin Yao, Qin Jiang and Cong Cao.

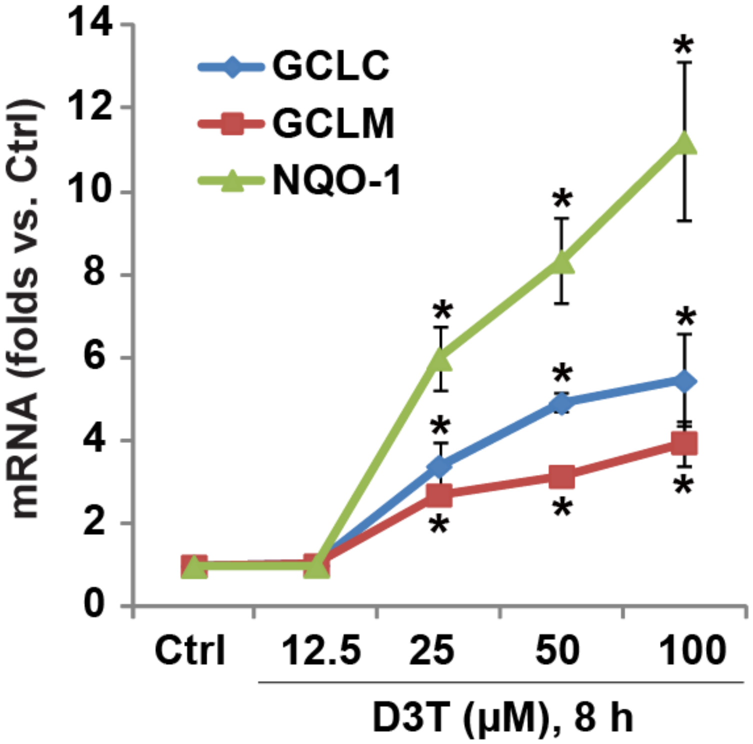

Supplementary Fig. 2. Relative mRNA expression of listed genes in D3T-treated APRE-19 cells was shown (**A**). ****p***<0.05 vs. Ctrl.

Supplementary Fig. 3. of "*3H-1,2-dithiole-3-thione protects retinal pigment epithelium cells against Ultra-violet radiation via activation of Akt-mTORC1-dependent Nrf2-HO-1 signaling*" by Ke-ran Li, Su-qing Yang, Yi-qing Gong, Hong Yang, Xiu-miao Li, Yu-xia Zhao, Jin Yao, Qin Jiang and Cong Cao.

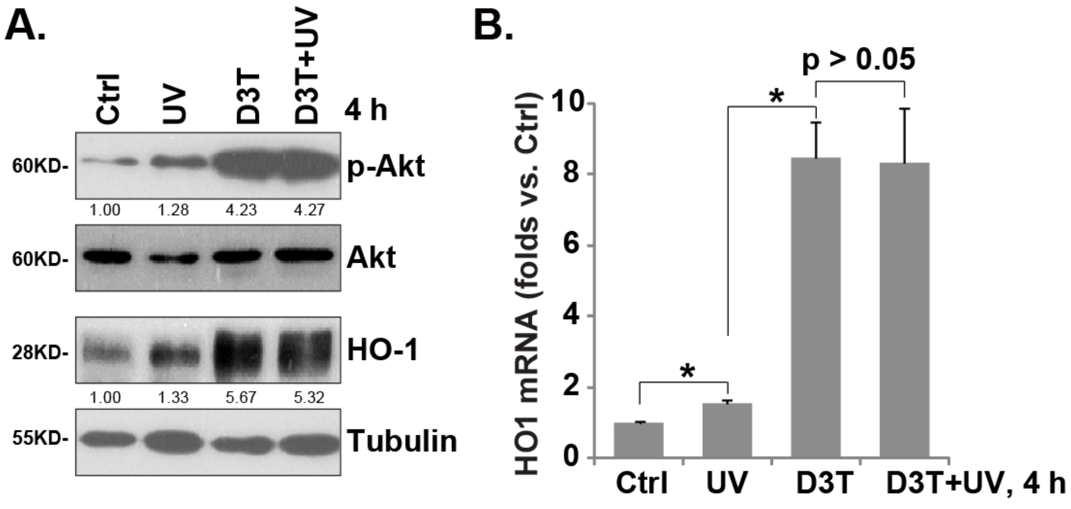

Supplementary Fig. 3. APRE-19 cells were irradiated with UV (30 mJ/cm^2) with or without D3T (50 μM, 30 min pretreatment), cells were further cultured for 4 h. Expressions of listed proteins were tested by Western blots (**A**), Akt phosphorylation and HO-1 protein expression were quantified (**A**). Relative HO-1 mRNA expression was examined by Real-time PCR assay (**B**). Experiments were repeated three times to insure consistency of results. **p*<0.05.
